# DynDSE: Automated Multi-Objective Design Space Exploration for Context-Adaptive Wearable IoT Edge Devices

**DOI:** 10.3390/s20216104

**Published:** 2020-10-27

**Authors:** Giovanni Schiboni, Juan Carlos Suarez, Rui Zhang, Oliver Amft

**Affiliations:** Digital Health, FAU Erlangen-Nürnberg, 91052 Erlangen, Germany; juan.c.suarez@fau.de (J.C.S.); rui.rui.zhang@fau.de (R.Z.); oliver.amft@fau.de (O.A.)

**Keywords:** health monitoring, automatic dietary monitoring, physiological sensing, pattern spotting, energy saving, embedded machine learning

## Abstract

We describe a simulation-based Design Space Exploration procedure (DynDSE) for wearable IoT edge devices that retrieve events from streaming sensor data using context-adaptive pattern recognition algorithms. We provide a formal characterisation of the design space, given a set of system functionalities, components and their parameters. An iterative search evaluates configurations according to a set of requirements in simulations with actual sensor data. The inherent trade-offs embedded in conflicting metrics are explored to find an optimal configuration given the application-specific conditions. Our metrics include retrieval performance, execution time, energy consumption, memory demand, and communication latency. We report a case study for the design of electromyographic-monitoring eyeglasses with applications in automatic dietary monitoring. The design space included two spotting algorithms, and two sampling algorithms, intended for real-time execution on three microcontrollers. DynDSE yielded configurations that balance retrieval performance and resource consumption with an F1 score above 80% at an energy consumption that was 70% below the default, non-optimised configuration. We expect that the DynDSE approach can be applied to find suitable wearable IoT system designs in a variety of sensor-based applications.

## 1. Introduction

**Figure 1:**
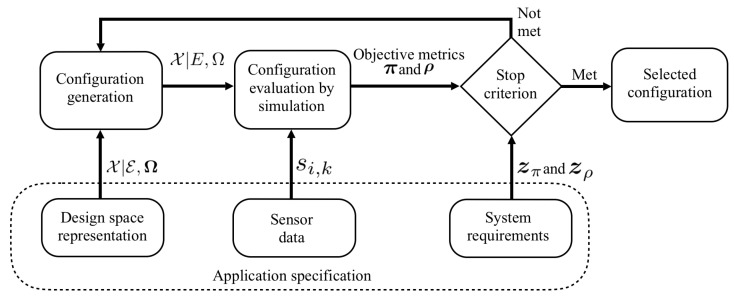
DynDSE procedure for wearable IoT edge devices. X|E,Ω: design space; X|E,Ω: system configuration; π benefit metrics set; ρ: cost metrics set; $z_{\pi }$: benefit requirement set; $z_{\rho }$: cost requirement set; $s_{i,k}$: data sample *i* from channel *k*.

Autonomous wearable IoT devices are being used for physiological and behavioural health-monitoring [1] and provide relevant health status information to their wearers [2,3]. Miniaturised electronics embedded in wearable accessories, garments, etc., provide the resources to retrieve pattern events from streaming sensor data and to interact with the wearer, which led to the concept of edge computing [4]. Edge computing aims to process data at the devices end, rather than the cloud to reduce network load and service response time. Furthermore, reducing communication bandwidth often lowers energy consumption, as well as privacy, and security concerns. For example, in medical IoT monitoring applications [5], a device may retrieve relevant events using embedded machine learning methods, thus sending only abstract event information to the cloud. Nevertheless, resource constrains are a key feature of IoT devices. A wearable IoT device typically consists of multiple sensors, a microcontroller (μC), which runs data processing algorithms, memory, and a radio module for data communication (Figure 1). Therefore, the optimisation of resource-constrained IoT edge devices has high priority in the design process [6].

The design of an IoT device can be interpreted as the selection of an optimal hardware and software configuration from the wide design space of possible options. Certain system configurations may be not compatible with specific system requirements, e.g., energy saving and retrieval performance.

Available μCs present differences in terms of resource consumption, execution time, and energy consumption. With the complex interplay of hardware and software components, it is a difficult task to quantify resource use and to manually identify optimal configurations that fulfil system requirements best. The size of the architectural design space often makes manual exploration of embedded systems unfeasible. Automated Design Space Exploration (DSE) [7,8] provides a computational framework to identify optimal configurations.

The design problem is exacerbated when the system does include functions that cannot be statically approximated, and by those with a dynamic effect on the resource-performance trade-off. Sampling strategies, e.g., context-adaptive sampling [9], are a basic dynamic function of wearable IoT devices. A context-adaptive sampling is a dynamic sampling strategy that tunes the sensor’s sampling rate based on a context measure, thus aiming at minimising energy consumption. The stochastic and variable nature of human behavioural patterns induces variability into context-adaptive sampling behaviour, which, in turn, drastically affects the resource-perfomance trade-off. Therefore, the main challenge for the design of a context-adaptive wearable IoT device lies in the identification of viable configurations that fulfil the system requirements under dynamically varying conditions. With DynDSE, we explicitly incorporate context-adaptive system behaviour in the design exploration and simulate systems with actual sensor data.

In this paper, we provide the following contributions:

1. We present a simulation-based Design Space Exploration (DynDSE) procedure for wearable IoT devices that employ context-adaptive pattern recognition algorithms for event retrieval, see Figure 1. We provide a formal characterisation of the design space, given a set of system functionalities, components, and their parameters. An iterative search evaluates configurations according to system requirements. The inherent trade-off embedded in conflicting objectives are explored to find an optimal configuration.
2. We perform a wearable IoT application evaluation to analyse the resource-performance trade-off considering static and dynamic design aspects through simulations with actual data of Electromyography (EMG)-monitoring eyeglasses in automated dietary monitoring.

In the present work, we formally introduce the DynDSE exploration framework in Section 3 and relevant optimisation metrics in Section 4. Subsequently, a comprehensive wearable IoT application case is presented and analysed in Section 5 and Section 6 to detail the potential of DynDSE.

## 2. Related Work

DSE frameworks are used for hardware/software co-design of heterogeneous multiprocessor and system-on-chip architectures [10], embedded systems [11], or Field Programmable Gate Array (FPGA) platforms [12]. In conventional DSE approaches, multiple metrics, e.g., energy consumption, memory demand, and cost, must be optimised concurrently according to some application requirements. The conflicting nature of objectives, which reflect many system characteristics, produces trade-offs inherent to the overall system performance. A decision on the most adequate system configuration needs to be taken according to a multi-objective optimisation process. The majority of DSE methods belongs to one of three analysis and evaluation categories, i.e., prototype-based, analytics-based, and simulation-based. The three categories differ in terms of a design time-modelling accuracy trade-off [8]. For example, the prototype-based evaluation provides adequate modelling accuracy, but requires development time with limited exploration capability. The analytics-based evaluation relies on analytical description of component interactions, which allows designers to explore larger design space portions in acceptable time. However, especially with complex architectures, the modelling accuracy of analytics-based evaluations is limited. The simulation-based evaluation is the most versatile approach, as time-modelling accuracy trade-offs of different designs can be achieved by tuning the simulation characteristics. For example, a lower abstraction simulation level, i.e., simulating digital signals between registers and combinational logic, yields higher accuracy but lowers simulation time for analysing software stacks. A higher abstraction simulation level, i.e., simulating system components at the cycle level, computes more efficient simulation, at the cost of averaging inter-cycle system state information. Moreover, simulation-based DSE enables dynamic profiling at run time, which allows the designer to quantify and optimise complex dynamic component interactions and workload.

In last two decades, many DSE approaches have been proposed to design wearable devices. For example, Bharatula et al. [13] proposed a design method to achieve a resource-performance trade-off for a highly-miniaturised multi-sensor context recognition system. Their evaluation showed that, through variations of system design space, the optimisation method was able to extend the battery lifetime by several hours. The same research group introduced multiple metrics to analyse the resource-performance trade-off of a wearable system, i.e., an accelerometer, a microphone, a light sensor, and a TI MSP430F1611 μC [14]. The authors presented an experimental validation in which a manual multi-objective optimisation was applied to find the best system configuration. In contrast to the works of Bharatula et al., we propose an analytical characterisation of the system architecture with the aim to automate the DSE modelling. Anliker et al. [15,16] presented an automatic design methodology based on abstract modules and task-dependent constraint sets. A multi-objective function incorporated recognition accuracy, energy consumption, and wearability, applied to classification of three modes of locomotion. In contrast, our simulation-based analysis is based on a realistic dataset collected in daily living. We evaluated the relevant effects of the free-living settings on the metrics. Beretta et al. [17] presented a model-based design optimisation to analytically characterise the energy consumption of a wearable sensor node. The authors described a multi-objective exploration algorithm to evaluate system configurations and relative trade-off. The method was application-driven with a fixed system architecture. Stäger et al. [18] took in account several system configuration options, including sensor types, sensor parameters, features, and classifiers. A case study was described related to detection of interactions with household appliances by means of a wrist worn microphone and accelerometer. Evidences were presented for an improvement in battery lifetime by a factor 2–4 with only little degradation in recognition performance.

The aforementioned DSE approaches focused on the evaluation and exploration of wearable device architectures under static workloads. However, todays wearable systems may adopt opportunistic sensing strategies to balance energy consumption and information acquired by a wearable or mobile systems. For example, Rault et al. [19] provided an analysis of techniques for energy consumption reduction in wearable sensors for healthcare applications. Opportunistic sensing strategies consider dynamic effects on the resource consumption trade-off, not considered in a static DSE. For example, an adaptive sampling scheme may reduce sampling rate according to a detected lower signal entropy. For instance, Mesin [20] proposed an adaptive sampling scheme based on sample prediction, where a non-uniform schedule increased the sampling rate only during bursts of physical activity. A multi-layer perceptron predicted subsequent samples and their uncertainties, triggering a measurement when the uncertainty of the prediction exceeded a threshold. In contrast, our approach employs a transparent state-based reactive model to estimate relevance of future samples. Scarabottolo et. al. [21] presented a dynamic sampling strategy for low-power embedded devices. The sampling rate tuning was based on the analysis of the signal’s spectral content. Rieger and Taylor [22] proposed a low-power analog system for real-time adaptive sampling rate tuning, proportional to the signal curvature. Different from our approach, a-priori knowledge was required. Moreover, in contrast to Rieger and Taylor’s [22] low-power analog system, we consider a pattern spotting problem to analyse performance that is frequently required in wearable IoT systems.

## 3. Exploration Framework

### 3.1. Design Space Representation

The design space configurations consist of set X of functionalities, realised by a set E of components, which are characterised by the set Ω of parameters. An example is provided at the end of this section.

Formally, the set of functionalities X is expressed as:

(1) $$\begin{array}{c} \begin{array}{cc} \; & X={\left\{\Xi_{\xi }\right\}}_{\xi \in \xi },\\ \; & \xi =\{\xi |\xi \in N_{1},\xi \leq N_{\Xi }\}, \end{array} \end{array}$$

where the functionality $\Xi_{\xi }$ is the element of the set X, ξ is the index set to X, and $N_{\Xi }$ is the number of elements of ξ.

A functionality $\Xi_{\xi }$ is carried out by one or more components grouped in the set $\epsilon_{\xi }$, indexed by the index set $q_{\xi }$, expressed as:

(2) $$\begin{array}{c} \begin{array}{cc} \; & \epsilon_{\xi }={\left\{\epsilon_{\xi ,q}\right\}}_{q\in q_{\xi }}\mathrm{and}\\ \; & q_{\xi }=\left\{q|q\in N_{1},q\leq N_{\xi }\right\}, \end{array} \end{array}$$

where the component $\epsilon_{\xi ,q}$ is the element of the set $\epsilon_{\xi }$, $q_{\xi }$ is the index set to $\epsilon_{\sim }$, and $N_{\xi }$ is the number of components associated to the functionality ξ.

Overall, the design space consists of a collection E of system components sets, indexed by the collection Q of index sets, expressed as:

(3) $$\begin{array}{c} \begin{array}{cc} \; & E={\left\{\epsilon_{\xi }\right\}}_{\xi \in \xi }\;\mathrm{and}\\ \; & Q=\{q_{\xi }|\xi \in \xi \}. \end{array} \end{array}$$

A component $\epsilon_{\xi ,q}$ is characterised by one or more component parameters grouped in the set $\omega_{\xi ,q}$, indexed by the index set $w_{\xi ,q}$, expressed as:

(4) $$\begin{array}{c} \begin{array}{cc} \; & \omega_{\xi ,q}={\left\{\omega_{\xi ,q,w}\right\}}_{w\in w_{\xi ,q}}\;\mathrm{and}\\ \; & w_{\xi ,q}=\left\{w|w\in N_{1},w\leq N_{\xi ,q}\right\}, \end{array} \end{array}$$

where the component parameter $\omega_{\xi ,q,w}$ is the element of the set $\omega_{\xi ,q}$, $w_{\xi ,q}$ is the index set to $\omega_{\xi ,q}$, and $N_{\xi ,q}$ is the number of component parameters associated to the functionality ξ and the component *q*. Overall, the design space consists of a collection Ω of component parameters sets, indexed by the collection W of index sets, expressed as:

(5) $$\begin{array}{c} \begin{array}{cc} \; & \Omega ={\left\{\omega_{\xi ,q}\right\}}_{\left(\xi ,q\right)\in {⋃}_{\xi \in \xi }\left(\xi \times q_{\xi }\right)}\;\mathrm{and}\\ \; & W=\{w_{\xi ,q}|\left(\xi ,q\right)\in \underset{\xi \in \xi }{⋃}\left(\xi \times q_{\xi }\right)\}. \end{array} \end{array}$$

For example, a spotting algorithm $\Xi_{1}$ is represented by a component $\epsilon_{1,1}$, e.g., a FFT-based algorithm, characterised by a component parameter $\omega_{1,1,1}$, e.g., the data frame size. Data sampling $\Xi_{2}$ is represented by a component $\epsilon_{2,1}$, e.g., an uniform sampling. A processing unit $\Xi_{3}$ is represented by a component $\epsilon_{3,2}$, e.g., a Texas Instrument μC, characterised by a component parameter $\omega_{3,2,1}$, e.g., the μC’s clock frequency. In a compact form, the design space is expressed as:

(6) X|E,Ω.

### 3.2. Configuration Generation

The configuration generation selects a design candidate to be evaluated in the simulation, i.e., see Section 3.3. The configuration generation is composed by two main stages. In the first stage, for each functionality $\Xi_{\xi }$, a component set $\epsilon_{\xi }^{c}$, indexed by the index set $q_{\xi }^{c}$, is selected as:

(7) $$\begin{array}{c} \begin{array}{cc} \; & \epsilon_{\xi }^{c}={\left\{\epsilon_{\xi ,q}\right\}}_{q\in q_{\xi }^{c}}\;\mathrm{and}\\ \; & q_{\xi }^{c}\subseteq q_{\xi }, \end{array} \end{array}$$

where $q_{\xi }^{c}$ represents a subset of the the index set $q_{\xi }$. Overall, a configuration consists of a collection *E* of system components sets, indexed by the collection $Q^{c}$ of index sets, expressed as:

(8) $$\begin{array}{c} \begin{array}{cc} \; & E={\left\{\epsilon_{\xi }^{c}\right\}}_{\xi \in \xi }\;\mathrm{and}\\ \; & Q^{c}=\left\{q_{\xi }^{c}|\xi \in \xi \right\}, \end{array} \end{array}$$

In the second stage, for each component $\epsilon_{\xi ,q}\in \epsilon_{\xi }^{c}$ a component parameters set $\omega_{\xi ,q}^{c}$, indexed by the index set $w_{\xi ,q}^{c}$, is selected as:

(9) $$\begin{array}{c} \begin{array}{cc} \; & \omega_{\xi ,q}^{c}={\left\{\omega_{\xi ,q,w}\right\}}_{w\in w_{\xi ,q}^{c}}\;\mathrm{and}\\ \; & w_{\xi ,q}^{c}\subseteq w_{\xi ,q}, \end{array} \end{array}$$

where $w_{\xi ,q}^{c}$ represents a subset of the the index set $w_{\xi ,q}^{c}$. Overall, a system configuration consists of a collection Ω of system component parameters sets, indexed by the collection $W^{c}$ of index sets, expressed as:

(10) $$\begin{array}{c} \begin{array}{cc} \; & \Omega ={\left\{\omega_{\xi ,q}^{c}\right\}}_{\left(\xi ,q\right)\in {⋃}_{\xi \in \xi }\left(\xi \times q_{\xi }^{c}\right)}\;\mathrm{and}\\ \; & W^{c}=\left\{w_{\xi ,q}^{c}|\left(\xi ,q\right)\in \underset{\xi \in \xi }{⋃}\left(\xi \times q_{\xi }^{c}\right)\right\}. \end{array} \end{array}$$

In a compact form, a system configuration is expressed as:

(11) X|E,Ω⊆X|E,Ω.

### 3.3. Configuration Evaluation

A metric estimates benefits π or costs ρ of a configuration. The configuration evaluation is based on two sets of metrics. The benefit metric set is defined as:

(12) $$\begin{array}{cc} \; & \pi ={\left\{\pi^{p}\left(X|E,\Omega \right)\right\}}_{p\in p}\;\mathrm{with} \end{array}$$

(13) $$\begin{array}{cc} \; & p=\{p|p\in N_{1},p\leq N_{p}\}, \end{array}$$

where the element $\pi^{p}\left(X|E,\Omega \right)$ is a benefit metric and $N_{p}$ is the number of benefit metrics. The benefit metric set π is subjected to the benefit requirement set as:

(14) $$\begin{array}{c} z_{\pi }={\left\{z_{\pi }^{p}\right\}}_{p\in p}, \end{array}$$

Similarly, the cost metric set is defined as:

(15) $$\begin{array}{cc} \; & \rho ={\left\{\rho^{r}\left(X|E,\Omega \right)\right\}}_{r\in r}\;\mathrm{with} \end{array}$$

(16) $$\begin{array}{cc} \; & r=\{r|r\in N_{1},r\leq N_{r}\}. \end{array}$$

where the element $\rho^{r}\left(X|E,\Omega \right)$ is a cost metric and $N_{r}$ is the number of cost metrics. The cost objective set ρ is subjected to the cost requirement set as:

(17) $$\begin{array}{c} z_{\rho }={\left\{z_{\rho }^{r}\right\}}_{r\in r}. \end{array}$$

Each $\pi^{p}\left(X|E,\Omega \right)$ and $\rho^{r}\left(X|E,\Omega \right)$ maps a configuration X|E,Ω to the real space IR, i.e., X|E,Ω→IR.

The overall optimisation process is formally described as follows. Given a design space X|E,Ω, the goal of DynDSE is to find the configuration X|E,Ω, which maximises benefits π and minimises costs ρ, while respecting the respective set of requirements. The problem can be interpreted as a constrained multi-objective optimisation:

(18) $$\begin{array}{c} \begin{array}{cc} \max_{X|E,\Omega \subseteq X,E|\Omega }\;\; & \sum_{p}\pi^{p}\left(X|E,\Omega \right)-\sum_{r}\rho^{r}\left(X|E,\Omega \right)\\ \; & s.t.\;z_{\rho }^{r}\leq 0\;\;\forall r\in r,\\ \; & \;\;\;\;z_{\pi }^{p}\geq 0\;\;\forall p\in p. \end{array} \end{array}$$

The optimisation provides a set of mutually conflicting solutions, which reflects the trade-offs in the design. To define optimality, one can usually exploit the concept of Pareto-dominance, i.e., a decision maker prefers a configuration to another if it is equal or better in all objectives and strictly better in at least one. As Künzli et al. [23] pointed out, several approaches exists to solve the multi-objective optimisation, e.g., exploration by hand [24], exhaustive search [25], or reduction to a single objective [26], as done in this work.

## 4. Optimisation Metrics

Table 1 lists the metrics introduced in this Section and their respective requirements.

### 4.1. Retrieval Performance Metric

The retrieval performance of an event retrieval algorithm is expressed through Precision-Recall metric [27], as follows:

(19) $$\begin{array}{c} \begin{array}{c} \mathrm{Precision}\;P=\pi^{1}\left(X|E,\Omega \right),\\ \mathrm{Recall}\;R=\pi^{2}\left(X|E,\Omega \right). \end{array} \end{array}$$

From the application perspective, the algorithm must be able to keep an adequate level of retrieval performance. The retrieval performance requirements is usually defined by expert knowledge and we refer to it as $z_{\pi }^{1}$ and $z_{\pi }^{2}$.

### 4.2. Execution Time Metric

A measure of computational complexity denotes the execution time of an algorithm, by pairing the algorithm module and the processing module. An algorithm’s abstraction is the decomposition of an algorithm in distinct stages, in which each stage is composed by one or more functions. Each function *f* is broken down by counting additions and subtractions (Add), multiplications (Mult), divisions (Div), square roots (Root), exponentials (Exp), and comparisons (Comp).

The number of machine cycles $n_{f}^{cyc}$ to compute a function *f* on a μC is defined as:

(20) $$\begin{array}{c} \begin{array}{cc} n_{f}^{cyc}= & \left(n_{f,\mathrm{Add}}^{op}\times n_{\mathrm{Add}}^{cyc}\right)+\left(n_{f,\mathrm{Mult}}^{op}\times n_{\mathrm{Mult}}^{cyc}\right)+\\ \; & \left(n_{f,\mathrm{Div}}^{op}\times n_{\mathrm{Div}}^{cyc}\right)+\left(n_{f,\mathrm{Root}}^{op}\times n_{\mathrm{Root}}^{cyc}\right)+\\ \; & \left(n_{f,\mathrm{Exp}}^{op}\times n_{\mathrm{Exp}}^{cyc}\right)+\left(n_{f,\mathrm{Comp}}^{op}\times n_{\mathrm{Comp}}^{cyc}\right), \end{array} \end{array}$$

where $n_{f,x}^{op}$ is the number of executions related to an arithmetical operation *x* to compute a function *f* and $n_{x}^{cyc}$ is the number of cycles to execute an arithmetical operation *x* on a μC. To estimate the execution time ${\mathrm{ET}}_{f}$ of a function *f*, the number of machine cycles $n_{f}^{cyc}$ is divided by the clock frequency ν of the μC, as follows:

(21) $${\mathrm{ET}}_{f}=\frac{n_{f}^{cyc}}{\nu }.$$

The definition of the execution time ET depends on the runtime mode. When the runtime mode is real time, ET is computed as:

(22) $$\mathrm{ET}=\rho^{1}\left(X|E,\Omega \right)=\sum_{f}{\mathrm{ET}}_{f}.$$

When the runtime mode is online, ET is computed as:

(23) $$\mathrm{ET}=\sum_{fr}\sum_{f}{\mathrm{ET}}_{f},$$

where fr is a data frame process. We refer to the system requirements for ET as $z_{\rho }^{1}$.

### 4.3. Energy Consumption Metric

**μC energy consumption**: Most μCs support an active state and a stand-by state. The average energy consumption in active state ${\mathrm{EC}}_{f}^{\mu C}$, related to the computation of a function *f*, is proportional to the ${\mathrm{ET}}_{f}$ and defined as:

(24) $${\mathrm{EC}}_{f}^{\mu C}={\mathrm{ET}}_{f}\times P_{\mathrm{act}}^{\mu C},$$

where ${\mathrm{EC}}_{f}^{\mu C}$ is expressed in Wh, $P_{\mathrm{act}}^{\mu C}$ is the power consumption of the μC in active mode expressed in W, and ${\mathrm{ET}}_{f}$ is expressed in hours.

The average energy consumption in stand-by state ${\mathrm{EC}}_{\mathrm{stb}}^{\mu C}$ is modelled as:

(25) $${\mathrm{EC}}_{\mathrm{stb}}^{\mu C}=T_{\mathrm{stb}}\times P_{\mathrm{stb}}^{\mu C},$$

where $T_{\mathrm{stb}}$ is the time period of inactivity expressed in hours, and $P_{\mathrm{stb}}^{\mu C}$ is the power consumption in stand-by mode expressed in W.

The μC energy consumption was calculated as:

(26) $${\mathrm{EC}}^{\mu C}=\sum_{fr}\sum_{f}{\mathrm{EC}}_{f}^{\mu C}+{\mathrm{EC}}_{\mathrm{stb}}^{\mu C},$$

where $\sum_{fr}\sum_{f}{\mathrm{EC}}_{f}^{\mu C}$ denotes the active state energy.

**Sensor energy consumption**: The average instantaneous energy consumption ${\mathrm{EC}}_{t}^{s}$ for a single sensing component is computed by applying the following equations:

(27) $$\begin{array}{cc} \; & I_{t}^{s}=I_{\mathrm{act}}^{s}\times D_{t}+I_{\mathrm{stb}}^{s}\times \left(1-D_{t}\right),\\ \; & {\mathrm{EC}}_{t}^{s}=I_{t}^{s}\times V\times t_{r}, \end{array}$$

where $I_{\mathrm{act}}^{s}$ is the sensor’s average current in active state, $I_{\mathrm{stb}}^{s}$ is the sensor’s average current in stand-by state, $D_{t}$ is the instantaneous duty cycle rate, ${\mathrm{EC}}_{t}^{s}$ is expressed in Wh, *V* is the voltage level of the sensing component, and $t_{r}$ is the temporal resolution expressed in hours.

The sensor energy consumption was calculated as:

(28) $${\mathrm{EC}}^{s}=\sum_{t}{\mathrm{EC}}_{t}^{s}.$$

**fFlash/Non-Volatile Memory Energy Consumption**: To estimate the flash and programmable memory, we formulated an energy model inspired by Konstantakos et al. [28]. Writing energy consumption was determined by:

(29) $${\mathrm{EC}}_{w}^{m}=\sum_{b}I_{\mathrm{write}}^{m}\times V\times t_{w},$$

where *b* indicates a block, $I_{\mathrm{write}}^{m}$ is the average current consumption in writing mode, and write time $t_{w}$ is the time required to write a memory block. The energy required to read a memory block was neglected. A static memory energy consumption term was computed as:

(30) $${\mathrm{EC}}_{s}^{m}=I_{\mathrm{stb}}^{m}\times V\times \left(T-\sum_{b}t_{w}\right),$$

where $I_{b}^{m}$ is the stand-by state current value, and *T* is the total simulation time.

The memory energy consumption was calculated as:

(31) $${\mathrm{EC}}^{m}={\mathrm{EC}}_{w}^{m}+{\mathrm{EC}}_{s}^{m}.$$

**Radio transmission energy consumption measure**: To estimate the energy consumption of the wireless communication, we relied on the energy model described by Prayati et al. [29]. The model considered the following three stages for transmission: (1) initialisation of transmission and transferring of the frame data from memory to the radio chip FIFO buffer, (2) back-off timeout, and (3) packet transmission via the wireless channel.

In order to calculate the energy consumption to transmit a packet, the following formula was applied:

(32) $$EC_{p}^{r}=I_{\mathrm{trans}}\times V\times t_{\mathrm{trans}},$$

where $I_{\mathrm{trans}}$ is the transmission current, *p* indicating packets, and $t_{\mathrm{trans}}$ is the time requested to prepare and send a packet. As $EC_{p}^{r}$ is expressed in Wh, $t_{\mathrm{trans}}$ must be converted in hours. In this work we considered $I_{\mathrm{trans}}=21.7$ mA, when a transmission power threshold 0 dBm is chosen, and $t_{\mathrm{trans}}=16\;\mathrm{ms}$ is the time requested to prepare and send a packet size of 114 Bytes.

The radio transmission energy consumption was calculated as:

(33) $${\mathrm{EC}}^{r}=\sum_{p}EC_{p}^{r}.$$

**Total energy consumption**: The energy consumption metric EC is computed as follows:

(34) $$\begin{array}{c} \begin{array}{cc} \; & \mathrm{EC}=\rho^{2}\left(X|E,\Omega \right)=\\ \; & {\mathrm{EC}}^{\mu C}+{\mathrm{EC}}^{s}+{\mathrm{EC}}^{m}+{\mathrm{EC}}^{r}. \end{array} \end{array}$$

The application context imposes an energy budget requirement to the behavioural monitoring. For example, while monitoring dietary behaviour, the wearable system should be able to work uninterruptedly for the entire day, in order to not miss relevant activities. The system requirement $z_{\rho }^{2}$ for EC indicates the energy required to deploy the application for the entire runtime. We defined $z_{\rho }^{2}$ as the quantity in mW calculated as:

(35) $$z_{\rho }^{2}=\frac{\mathrm{BC}}{\mathrm{RT}}\cdot \phi ,$$

where BC is the battery capacity in mWh, RT is the required runtime of the application expressed in hours, e.g., 16 h, and the factor 0<ϕ≤1 considers the effect of external factors, which can affect the battery life. In this work we considered ϕ=0.9.

### 4.4. Memory Demand Metric

The memory demand MD is an upper bound of the memory required by the system to execute an event retrieval. MD is computed by considering four terms, i.e., the code memory $m_{c}$, the data memory $m_{d}$, the processing memory $m_{f}$, and the event memory $m_{e}$.

The memory demand is defined as:

(36) $$\begin{array}{c} \begin{array}{cc} \; & \mathrm{MD}=\rho^{3}\left(X|E,\Omega \right)=m_{c}+m_{d}+\sum_{f}m_{f}+m_{e},\\ \; & \mathrm{with}\;m_{f}=m_{f,\mathrm{Int}}+m_{f,\mathrm{Float}},\\ \; & \mathrm{and}\;m_{e}=m_{e,\mathrm{Int}}+m_{e,\mathrm{Float}}, \end{array} \end{array}$$

where $m_{f,\mathrm{Int}}$ and $m_{e,\mathrm{Int}}$ are the memory required to store integer values, and $m_{f,\mathrm{Float}}$ and $m_{e,\mathrm{Float}}$ are the memory required to store float values. We refer to the system requirement for MD as $z_{\rho }^{3}$, which represents the maximum amount of memory available to store information on a certain μC.

### 4.5. Communication Latency Metric

The communication latency is the time span between the event-related raw sensor data measurements and the delivery moment at the receiver of the retrieved event information. The communication latency metric CL is computed as follows:

(37) $$\begin{array}{cc} \; & \mathrm{CL}=\rho^{4}\left(X|E,\Omega \right)=\sum_{f}{\mathrm{ET}}_{f}+\frac{m_{e}}{\nu_{tr}}, \end{array}$$

(38) $$\begin{array}{cc} \; & \mathrm{with}\;\nu_{tr}=\frac{\mathrm{MPS}}{connInterval}, \end{array}$$

where MPS is the transmitter’s maximum payload size, e.g., 216 bits for Bluetooth Low Energy (BLE), connInterval defines the time of connection events, i.e., ranges from 7.5 ms to 4.0 s with steps of 1.25 ms for BLE, and $\nu_{tr}$ is the transmission data rate. We refer to the system requirement for CL as $z_{\rho }^{4}$, which represents the communication latency tolerance for the application.

## 5. IoT Application Evaluation

### 5.1. Smart Eyeglasses to Monitor Eating in Free-Living

We implemented our DynDSE for the design optimisation of 3D-printed regular-looking eyeglasses, which accommodate processing electronics, EMG electrodes, antenna, and power supply. Smart eyeglasses are particularly suited for automated dietary monitoring to unobtrusively detect intake and eating events from activities around the head throughout everyday life, thus replacing classic food journaling and supporting disease management [30,31]. As typical wearable IoT devices, smart eyeglasses could process data locally and provide estimates to other body-worn devices, e.g. smartphones. Furthermore, the monitoring task is a typical example of wearable IoT applications in remote health assistance.

Two pairs of electrodes were symmetrically integrated at the eyeglasses frame, located around the temple ear bends. Contraction of temporalis muscles was monitored bilaterally resulting in two EMG signal channels.

The EMG sensor data stream was segmented into eating and non-eating periods by pattern spotting algorithms. The spotting algorithms were designed to extract features in continuous EMG sensor data and perform one-class classification to identify eating events (i.e., time span between the start and the end of an eating activity), see Section 5.2.1.

### 5.2. Design Space Representation

We considered $N_{\xi }=4$ system functionalities: $\Xi^{1}$, an algorithm for event retrieval, $\Xi^{2}$, a data sampling strategy, $\Xi^{3}$, a μC and $\Xi^{4}$, a runtime mode.

Table 2 presents the design space considered in this work. Our design space included two spotting algorithms, i.e., $\epsilon_{1,1}$ and $\epsilon_{1,2}$, which were considered for execution on three μCs, i.e., $\epsilon_{3,1},\epsilon_{3,2}$ and $\epsilon_{3,3}$. Moreover, two data sampling strategies, i.e., $\epsilon_{2,1}$ and $\epsilon_{2,2}$, were considered, while applying two runtime modes, i.e., $\epsilon_{4,1}$ and $\epsilon_{4,2}$.

To deal with the requirements in this case study, we relaxed the optimisation problem of Equation (18) into one of maximisation as follows:

(39) $$\begin{array}{c} \begin{array}{cc} \max_{X|E,\Omega \subseteq X|E,\Omega }\; & \sum_{p}\pi^{p}\left(X|E,\Omega \right)\\ \; & s.t.\;z_{\rho }^{r}\leq 1\;\;\forall r\in r,\\ \; & \;\;\;\;z_{\pi }^{p}\geq 1\;\;\forall p\in p. \end{array} \end{array}$$

The above constrained optimisation problem was solved by a grid search-based approach, evaluating a grid of possible configurations with an exhaustive search. At each iteration, a system configuration was generated and the sensor data processed through the simulation. The metrics were estimated and compared with the respective requirements.

#### 5.2.1. Algorithm ($\Xi_{1}$)

**FFT-based spotting:** The first pattern spotting method was introduced by Zhang and Amft [32] in order to identify eating moments. An online non-overlapping sliding window segmentation with length *m* expressed in seconds was used to extract features in continuous EMG data. A one-class classification was performed by a one-class SVM (oc-SVM). Details of the feature extraction and one-class classification can be found in [32]. The ocSVM was trained applying the leave-one-participant-out (LOPO) cross-validation strategy. Hyperparameter optimisation was performed using grid search approach.

**WPD-based spotting:** We designed the second pattern spotting method inspired by the classification task presented in [33]. An online non-overlapping sliding window segmentation with length *m* expressed in seconds was used. From each segmented frame, the maximum sample value was extracted and compared to a threshold experimentally found. When the sample value was lower than the threshold, the frame was not fed to the spotting pipeline. In the pre-processing module, the EMG signals were passed through a notch filter of 50 Hz to remove power line’s interferences, likely to occur in free-living, also de-trended by a digital high pass filter of 20 Hz and rectified. In the feature extraction module, the signal was passed through a Wavelet Packet Decomposition (WPD), to extract *c* features, i.e., the WPD coefficients, in the time-frequency domain. The depth level of the tree decomposition was kept constant, i.e., l=2. A principal component analysis (PCA) was subsequently used to reduce the number of features from *c* to *d*. After normalisation, the features were used as discriminant of the target class by using a ocSVM, with number of support vectors v=1500.

The ocSVM was trained applying the LOPO cross-validation strategy. Hyperparameter optimisation was performed using grid search approach.

Table 3 presents a breakdown of the spotting algorithms.

#### 5.2.2. Data Sampling ($\Xi_{2}$)

A context-adaptive sampling algorithm needs two main components, i.e., a context measure and a response model, to adapt the sampling rate depending on an estimation of relevance of future samples. As a context measure, we employed a basic representation of EMG signal energy. A feedforward state-based model that alternates between attentive and sleep states was adopted as response model. Our sampling strategy was based on the *n*-shots measure paradigm: the sensor wakes up, takes *n* samples, and goes to sleep again. The energy content from the *n* samples was used to compute the context measure as:

(40) $$\begin{array}{ccc} \; & e_{k}=\frac{\sum_{i=1}^{n}s_{i,k}}{n},\; & \theta_{t}=\max\left(e_{1},\ldots ,e_{k},\ldots ,e_{K}\right), \end{array}$$

where $e_{k}$ is the signal energy for the $k^{th}$ channel, $s_{i,k}$ is the $i^{th}$ value sampled from the *n*-shots measurement, $\theta_{t}$ is the context measure, t is the time-step, *K* is the number of channels that connect to the same system. A linear mapping function converted $\theta_{t}$ to a candidate duty cycle rate $D_{t+1}^{*}$:

(41) $$D_{t+1}^{*}=D_{l}+\left[\frac{D_{h}-D_{l}}{\theta_{h}-\theta_{l}}\cdot \theta_{t}-\theta_{l}\right],$$

where $D_{l}$ is the minimum duty rate set to $\theta_{l}$, which was estimated from the signals noise. A maximum duty rate $D_{h}$ was set to $\theta_{h}$. We adjusted the model’s sensitivity by tuning $\theta_{h}$.

The behaviour of the response model is described by Equation (42).

The computation of the duty cycle rate for the next period was based on a comparison between the candidate duty cycle rate $D_{t+1}^{*}$ and the threshold value $D_{TH}$.

As the $D_{t+1}^{*}$ exceeds the threshold $D_{TH}$, the response model switches from inattentive state to attentive state. The attentive state is characterised by a monotonically increasing duty cycle rate. As the $D_{t+1}^{*}$ drops below the threshold $D_{TH}$ and the attention time τ expires, the response model switches back from attentive state to inattentive state. The duty cycle rate’s decision rules for the two states were computed as follows:

(42) $$\begin{array}{cc} \left(1\right) & \;\mathrm{Inattentive}\;\mathrm{state}\;\\ \; & \mathrm{If}\;(D_{t+1}^{*}<D_{TH})\;\mathrm{and}\;\tau \;\mathrm{elapsed}):\\ \; & D_{t+1}=D_{t+1}^{*}\\ \left(2\right) & \;\mathrm{Attentive}\;\mathrm{state}\;\\ \; & \mathrm{If}\;(D_{t+1}^{*}>D_{TH}):\\ \; & D_{t+1}=\left\{\begin{array}{c} D_{t},\;\;D_{t+1}^{*}\leq D_{t}\\ D_{t+1}^{*},\;\;D_{t+1}^{*}>D_{t}. \end{array}\right. \end{array}$$

Table 4 presents a break down of the context-adaptive sampling algorithm. More details can be found in our previous work [34].

#### 5.2.3. μC ($\Xi_{3}$)

The processing module simulates the behaviour of an energy-efficient μC while computing the algorithm’s functions at a certain clock frequency. We considered three commonly widely used μCs, i.e., PSoC1 M8C, TI MSP430F1611, and ARM CortexM3. A Li-Ion polymer battery provided energy to the system. Each μC was provided with memory for local data processing, i.e., Flash and non-volatile memory. Table 5 shows the number of machine cycles $n_{x}^{cyc}$ related to the arithmetical operation *x* for different μCs.

#### 5.2.4. Runtime mode ($\Xi_{4}$)

Two runtime modes were considered in this work, i.e., online and real time. In our application, real time mode implies that as soon as a data frame has been recorded, the output of the data processing must be available. With online data processing, the temporal requirement is more relaxed, as the output of the data processing must be available by the end of the runtime.

### 5.3. Configuration Evaluation

#### 5.3.1. Sensor Dataset

Ten healthy volunteers (4 females, 6 males) aged between 20 and 30 years wore the EMG-monitoring eyeglasses for one day. The application data used in the simulation were sensor data collected at uniform sampling rate, i.e., 256 Hz. The eyeglasses were attached after getting up in the morning and kept on till bed time. When a risk of contamination with water existed, the participants were allowed to remove the eyeglasses. Participants manually logged the occurrence of eating events in a diet journal with a one minute resolution.

#### 5.3.2. Multi-Objective Computation

Our framework is based on a simulation which reproduces the functionalities of a wearable IoT system. The sensor’s and μC’s behaviour can be emulated with a finite-state machine approach, e.g., Buschhoff et al. [35], in order to reproduce dependencies between the system components and between hardware and software.

We evaluated the algorithm’s retrieval performance considering the total number of samples of all eating events according to ground truth labels, the total number of samples of all retrieved eating events, and the sum of the number of samples correctly retrieved belonging to eating events segments.

The number of machine cycles for an instance of a oc-SVM for the WPD-based spotting algorithm, with hyper-parameters (m=256,d=20), applying Equation (20), is computed as:

- Kernel SVM:
  $\left[v\cdot 60\right]+\left[2v\cdot 50\right]+\left[1\cdot 12\right]\approx 24\times 10^{4}$ cycles, where v=1500, is the number of support vectors;
- Radial basis function:
  $\left[v\left(2d-1\right)\cdot 60\right]+\left[v\left(d-1\right)\cdot 50\right]+\left[1\cdot 12\right]+\left[v\cdot 210\right]\approx 525\times 10^{4}$ cycles.

For simplicity, we assumed that all operations were performed using float data type.

Accordingly to Equation (21), the ${\mathrm{ET}}_{f}$ is computed from the number of machine cycles $n_{f}^{cyc}$, as inversely proportional to the clock frequency ν of a considered μC. Table 6 lists the clock frequencies of the three candidate μCs.

Time ${\mathrm{ET}}_{f}$ to execute the oc-SVM on a ARM CortexM3 was 114.3ms.

Accordingly to Equation (24), the ${\mathrm{EC}}_{f}^{\mu C}$ is computed multiplying the ${\mathrm{ET}}_{f}$ and the μC energy consumption $P_{\mathrm{act}}^{\mu C}$. The μC switching behaviour is regulated by the time constraints given by the ET and the sensor data frequency. Table 6 lists the current consumptions $I_{\mathrm{act}}^{\mu C}$ in active state, $I_{\mathrm{stb}}^{\mu C}$ in stand-by state, and the voltage level *V*, of the three candidate μCs. For example, the energy consumption ${\mathrm{EC}}_{f}^{\mu C}$ to execute an instance of the oc-SVM on a ARM CortexM3 was 0.732μWh.

**Figure 2:**
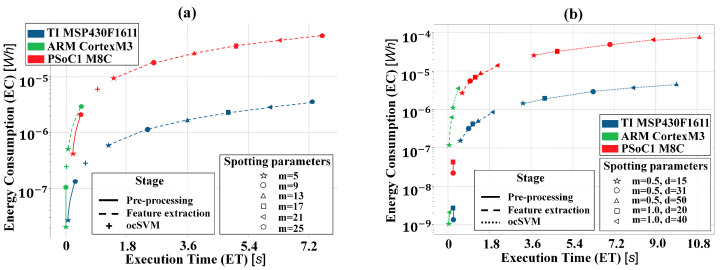
(**a**) FFT-based spotting: Relation between execution time and energy consumption. Some of the spotting parameters were omitted for readability. The spotting parameter *m* represents the data frame size. (**b**) WPD-based spotting. Relation between execution time and energy consumption. The spotting parameter *m* represents the data frame’s size and *d* represent the reduced feature space’s dimension.

Figure 2a,b show the linear relation between $\sum_{f}{\mathrm{ET}}_{f}$ and $\sum_{f}{\mathrm{EC}}_{f}^{\mu C}$, for the individual spotting stage κ. A log scale for the y axes was defined in order to make the plot more readable. A tuning effect of the spotting parameters on the execution time and on the energy consumption, is evident. The higher complexity of a parameter combination, the more machine cycles $n_{f}^{cyc}$, execution time ${\mathrm{ET}}_{f}$, and energy consumption ${\mathrm{EC}}_{f}$.

Table 5 and Table 6 list the memory specifications and the data resolution for the three candidate μCs. Each μC had a RAM memory and a larger flash support, and their capacity represented a system constraint.

By switching runtime mode, i.e., real-time mode or online mode, $m_{d}$ was defined as follows. In real-time mode, the $m_{d}$ was defined as the amount of data memory for a window size *m*. In online mode, the $m_{d}$ was estimated as the peak of memory footprint required to process the data stream continuously without data loss, using a ring buffer.

**Figure 3:**
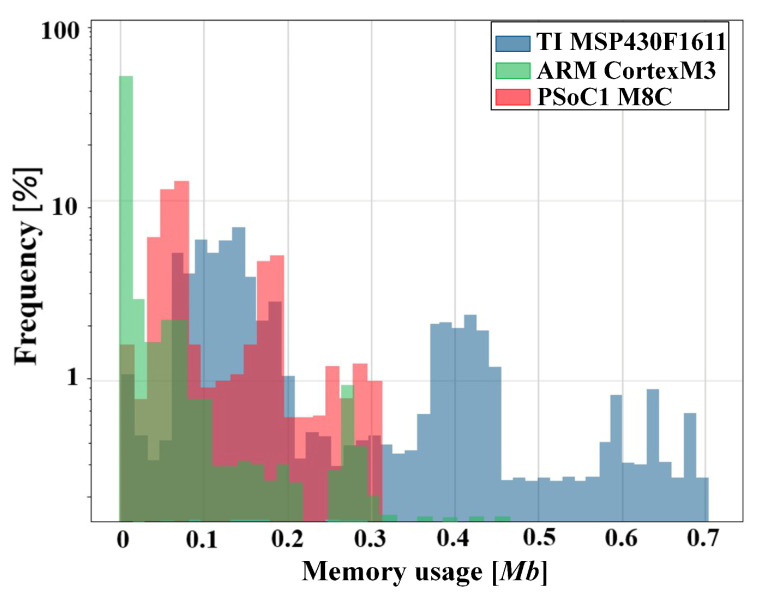
Memory $m_{d}$ foot-print for the online data buffer while executing the WPD-based spotting. Each colour is related to a specific μC.

Depending on their characteristics, the considered μCs had a certain latency that shaped the distribution of their memory footprint. For example, Figure 3 shows the distribution of the memory $m_{d}$ footprint related to a daylong processing. The BLE transmission’s specifications defined the maximum $\nu_{tr}$ as 305 kbps and the MPS as 215 bits [36]. Equation (38) defines the actual $\nu_{tr}$ by tuning the connInterval parameter, which ranged from 7.5 ms to 4.0 s with steps of 1.25 ms. For example, a retrieved eating event was represented by two time stamps, which represent the start and end of an eating event. A time stamp can be represented as an unsigned short variable type in 2 bytes (i.e., 5 bits for day, 5 bits for hour, and 6 bits for minute). The communication latency CL to deliver an event retrieved by the FFT-based spotting (m = 13) on a ARM CortexM3 was 886ms, according to Equation (37).

Considering the typical event frequencies in human daily behaviour and the negligible memory footprint for event information, communication latency was omitted from the following analyses.

#### 5.3.3. Multi-Objective Visualisation

To visualise whether the metrics lie within the system requirements’ boundary conditions or not, we exploited radar plots. Radar plots support the representation of the trade-off across the design space. Each y-axes is related to an objective, i.e., P, R, EC, ET, and MD, and normalised for the respective requirements, yielding a feasibility region within the unitary axes. Thus, a radar plot point beyond the unitary axes indicates unacceptable configurations. The unitary axes represents an upper bound regarding the requirements.

#### 5.3.4. Multi-Objective Analysis

For FFT-based, by tuning the parameter *m*, the precision’s variation ranged from 48.8% for m=1, to 95.7% for m=33. The recall R variation ranged from 62.9% for m=29, to 99.2% for m=1. The F1-score variation ranged from 63.3% for m=1, to 85.2% for m=9. For WPD-based, by tuning the parameters *m* and *d* the precision P variation ranged from 38.7% for m=0.25,d=50, to 95.7% for m=0.5,d=15. The recall R variation ranged from 52.7% for m=0.25,d=28, to 86.2% for m=1,d=40. The F1-score variation ranged from 49.1.0% for m=0.25,d=50, to 85.9% for m=1,d=40. The system requirements $z_{\pi }^{1}$ and $z_{\pi }^{2}$ are indicated as horizontal lines. It is evident that only a subset of spotting parameters respects the requirements.

**Figure 4:**
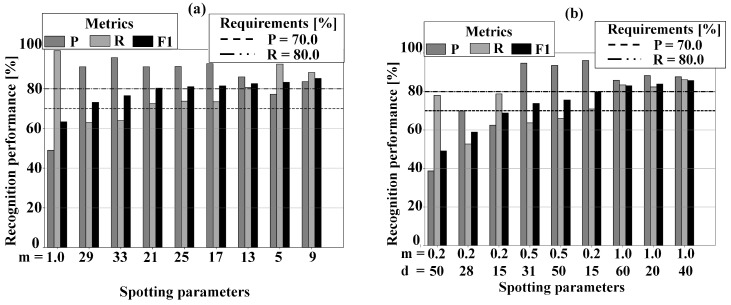
(**a**) FFT-based spotting. Average retrieval performance when varying the spotting parameters in uniform sampling mode. Bars were sorted by increasing retrieval performance. The spotting parameter *m* represents the data frame size. (**b**) WPD-based spotting. Average retrieval performance when varying the spotting parameters in uniform sampling mode. Bars were sorted by increasing retrieval performance. The spotting parameter *m* represents the data frame’s size and *d* represent the reduced feature space’s dimension.

**Figure 5:**
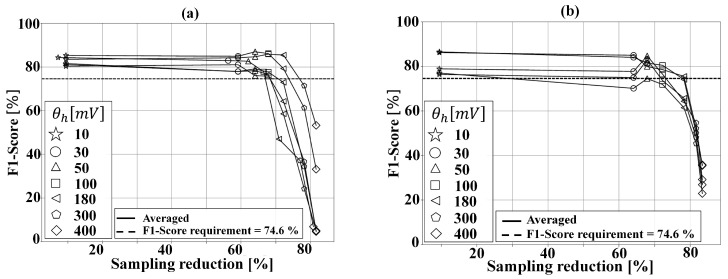
(**a**) FFT-based spotting. Average retrieval performance vs. sampling reduction when varying $\theta_{h}$. Individual lines correspond to the spotting parameters, which respect P and R requirements in Table 1. The F1-score requirement is derived by the same P and R requirements. The used parameters for the context-adaptive sampling were: $D_{h}=1$, $D_{l}=0.1$, $D_{TH}=0.6$, n=4, τ=3s, $\theta_{l}=10\;\mathrm{mV}$. (**b**) WPD-based spotting. Average retrieval performance vs. sampling reduction when varying $\theta_{h}$. Individual lines correspond to the spotting parameters that respect P and R requirements in Table 1. The F1-score requirement is derived from the same P and R requirements. The used parameters for the context-adaptive sampling were: $D_{h}=1$, $D_{l}=0.1$, $D_{TH}=0.6$, n=4, τ=3s, $\theta_{l}=10\;\mathrm{mV}$.

Figure 4a,b show the results of the cross-validated FFT-based and WPD-based spotting algorithms in uniform sampling mode, when tuning their spotting parameters. Specifically, precision P, recall R, and F1-score, are reported for all spotting parameters combinations. The degree of sampling reduction was changed by tuning $\theta_{h}$ in order to find a balance between retrieval performance and resource consumption. Figure 5a,b show the trade-off between F1-score and sampling reduction for the FFT-based and WPD-based spotting algorithms. It is clear that we can keep the retrieval performance up over 80% while performing a sampling reduction over 70%, in both methods. Comparing the two methods, the WPD-based spotting appears to be more robust against the down-sampling effect, presenting a lower degradation of retrieval performance for a given sampling reduction degree.

**Figure 6:**
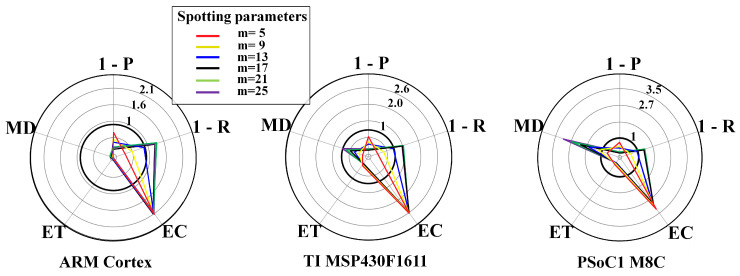
FFT-based spotting and uniform sampling. Resource-performance trade-off for real-time mode, including different μCs: ARM CortexM3 (**left**), TI MSP430F1611 (**center**), PSoC1 M8C (**right**). List of objectives: P = precision, R = recall, EC = energy consumption, ET = execution time, MD = memory demand.

**Figure 7:**
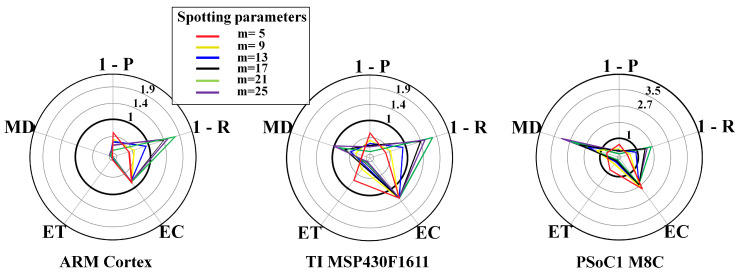
FFT-based spotting and context-adaptive sampling. Resource-performance trade-off for real-time mode, including different μCs: ARM CortexM3 (**left**), TI MSP430F1611 (**center**), PSoC1 M8C (**right**). List of metrics: P = precision, R = recall, EC = energy consumption, ET = execution time, MD = memory demand. Context-adaptive sampling parameters are: $D_{h}=1$, $D_{l}=0.1$, $D_{TH}=0.6$, n=4, τ=3s, $\theta_{l}=10\;\mathrm{mV}$, $\theta_{h}=180\;\mathrm{mV}$.

**Figure 8:**
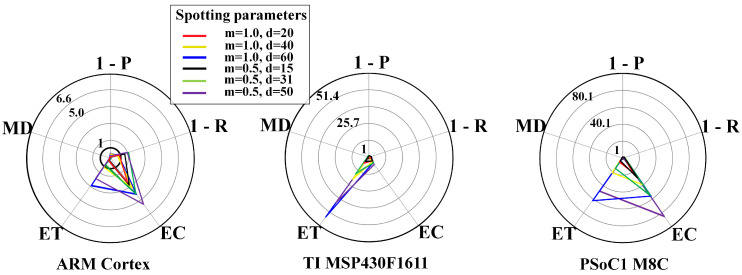
WPD-based spotting and uniform sampling. Resource-performance trade-off for real-time mode, including different μCs: ARM CortexM3 (**left**), TI MSP430F1611 (**center**), PSoC1 M8C (**right**). List of metrics: P = precision, R = recall, EC = energy consumption, ET = execution time, MD = memory demand.

**Figure 9:**
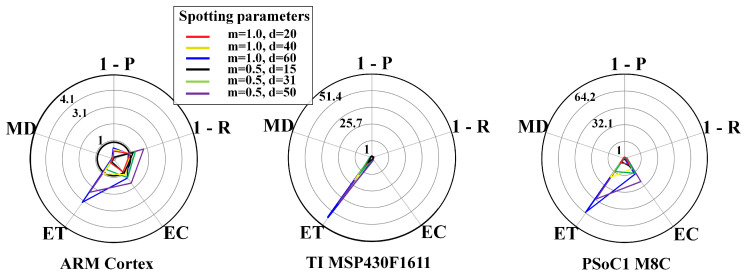
WPD-based spotting and adaptive sampling. Resource-performance trade-off for real-time mode, including different μCs: ARM CortexM3 (**left**), TI MSP430F1611 (**center**), PSoC1 M8C (**right**). List of metrics: P = precision, R = recall, EC = energy consumption, ET = execution time, MD = memory demand. The used parameters are: $D_{h}=1$, $D_{l}=0.1$, $D_{TH}=0.6$, n=4, τ=3s, $\theta_{l}=10\;\mathrm{mV}$, $\theta_{h}=180\;\mathrm{mV}$.

**Figure 10:**
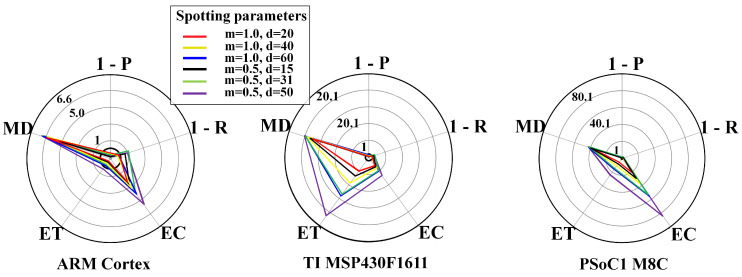
WPD-based spotting and uniform sampling. Resource-performance trade-off for online mode, including different μCs: ARM CortexM3 (**left**), TI MSP430F1611 (**center**), PSoC1 M8C (**right**). List of metrics: P = precision, R = recall, EC = energy consumption, ET = execution time, MD = memory demand.

**Figure 11:**
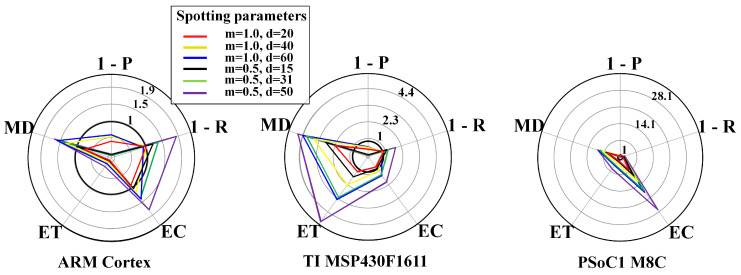
WPD-based spotting and context-adaptive sampling. Resource-performance trade-off for online mode, including different μCs: ARM CortexM3 (**left**), TI MSP430F1611 (**center**), PSoC1 M8C (**right**). List of metrics: P = precision, R = recall, EC = energy consumption, ET = execution time, MD = memory demand. The used parameters are: $D_{h}=1$, $D_{l}=0.1$, $D_{TH}=0.6$, n=4, τ=3s, $\theta_{l}=10\;\mathrm{mV}$, $\theta_{h}=180\;\mathrm{mV}$.

From Figure 6, Figure 7, Figure 8, Figure 9, Figure 10 and Figure 11, the trade-off across the metrics are shown for different system configurations. Figure 6 and Figure 7 show results for the FFT-based spotting in real-time for uniform and context-adaptive sampling. In uniform sampling, i.e., Figure 6, the largest requirement breach on any μC was due to the energy consumption EC. In context-adaptive sampling, i.e., Figure 7, the reduction of the energy consumption EC determined a set of feasible configurations on the ARM Cortex M3. In both sampling modalities, the execution time ET fulfilled the real-time requirements on any μC, although the memory demand MD compromised the feasibility of the configurations on the PSoC1 M8C.

Figure 8 and Figure 9 show results for the WPD-based spotting in real-time for uniform and context-adaptive sampling. In uniform sampling, i.e., Figure 8, the largest requirement breaches on any μC were due to the execution time ET and the energy consumption EC. In context-adaptive sampling, i.e., Figure 9, the reduction of the execution time ET determined a set of feasible configurations on the ARM Cortex M3. Overall, the memory demand MD was neglectable in all configurations, as only a data frame had to be stored.

Figure 10 and Figure 11 show results for the WPD-based spotting in online mode for uniform and context-adaptive sampling. The online mode required a more intensive memory use, due to the longer ring buffer, that in turn made the $z_{\rho }^{3}$ a more stringent requirement. In uniform sampling, i.e., Figure 10, the largest requirement breaches on any μC were due to the energy consumption EC and the memory demand MD. In context-adaptive sampling, i.e., Figure 11, the reduction of the energy consumption EC determined a set of feasible configurations on the ARM Cortex M3.

**Figure 12:**
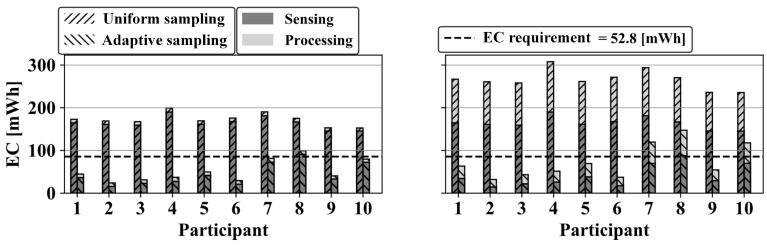
Estimated energy consumption (EC) for the individual participants on the TI MSP430F1611. **Left**: FFT-based spotting with m=13. **Right**: WPD-based spotting with (m=256,d=20).

Figure 12 depicts the energy consumption estimated from the best system configuration, related to the individual participants. For three participants, the boundary conditions are not respected, implying a reduced application’s runtime with respect to the system requirements.

## 6. Discussion

The simulation-based DynDSE presented here targets wearable IoT device design, which run time-variable recognition algorithms on the device. Processing a large volume of data locally and enabling local inference is key to a scalable IoT network. Furthermore, manual tuning of hardware and algorithms in a physical implementation is tedious. The DynDSE simulation-based approach can cover a wide configuration space to identify a balance between resources and event retrieval performance. Our case study demonstrated interactions and dependencies among hardware and algorithm components, and justified the need for co-designing and developing of associated functionalities. We chose two example retrieval algorithms to illustrate different effects on the optimisation result (cf. Figure 9, Figure 10, Figure 11 and Figure 12). FFT-based oc-SVM spotting was published before [32]. The WPD-based spotting showed performance improvements P and R, but also implications for execution time ET, thus further illustrating the design trade-off features of our methodology. The processing functionality ($\Xi_{3}$) affected the execution time ET, due to the μC type and speed, and the memory demand MD, due to its memory capacity. The μC’s energy consumption EC heavily depended on the algorithmic complexity, see Figure 12. Executing the WPD-based spotting, the μC’s energy consumption EC was comparable with the sensor’s energy consumption EC. As the computational complexity was shrunk by employing the FFT-based spotting, the energy consumption EC became neglectable. The algorithm ($\Xi_{1}$) and data sampling ($\Xi_{2}$) functionality were the most influencing configuration elements on the system’s metrics. Tuning the algorithm parameters affected precision P and recall R, and the required memory demand MD. Data sampling had the highest impact on the energy consumption EC and the tuning of the algorithm parameters had the lowest.

We found that our context-adaptive sampling strategy kept the performance of the spotting pipeline at a average F1-score over 80% while reaching almost 70% reduction in resource consumption.

DynDSE requirements in the application evaluation were set according to Table 1. While in our analysis, retrieval performances (P, R) larger than 80% were reached for optimised parameter settings, our requirements $z_{\pi }^{1}$ and $z_{\pi }^{2}$ followed literature recommendations [37] suggesting that even 70% in retrieval performance has relevant application value. Energy consumption requirement $z_{\rho }^{2}$ was set considering the capacity and size of standard lithium-ion batteries, and the application runtime, according to Equation (35). Execution time and memory demand requirements $z_{\rho }^{1}$ and $z_{\rho }^{3}$ were dictated by the algorithm and the μC characteristics, respectively. While the retrieved system configurations and their performances appear relevant, a direct comparison to prior work is not feasible, due to the diversity in analysis goals, applications, and dataset characteristics. First, many investigations optimise for a fraction of the DynDSE metrics only, e.g., recognition performance. Second, sensor and algorithm choice span a wide value space for performance metrics. Our investigation aimed at defining a generalisable procedure, which provides trade-off indicators across a variety of design space options and could thus assist designers in taking decisions and investigate details depending on application relevance.

Figure 7 and Figure 8 show the analysis of the variance in the resource-perfomance trade-off. Under the same P and R, higher sampling reduction can be achieved, which corresponds to lower energy consumption EC. In context-adaptive sampling mode, the resource consumption is proportional to the event frequency and the duration of event patterns. Consequently, for one configuration, resource saving varies according to individual behaviour. Population-averaged models do not guarantee to fulfil the system requirements for every individual. In our case study, a homogeneous study group of university students was included, however the resource consumption estimation did not respect the boundary conditions for all participants, as shown in Figure 12.

Personalising models increases computational complexity and entails more complicated deployment. A reasonable approach is to take into account the heterogeneity of the population by defining subpopulations having similar behaviour and include a safety margin.

We derived approximate machine cycle numbers, which limit accuracy of execution time estimation. The exact number of cycles is highly dependent on the algorithm implementation and compiler. Thus, our analysis could be integrated with extended target-dependent hardware and machine instruction simulators. Another source of inaccuracy are the energy consumption measures, as we did not consider overhead of the electronic circuits. We considered for simplicity only floating point operations. Differentiating between integer and floating point operations would result in higher modelling accuracy. Also, differentiating the range of variables and datatypes may improve the modelling.

The metric set serves as mapping between the design space and the application specifications, whose definition largely depends on the application requirements. Therefore, a direct comparison of metric outcomes is limited. However, depending on the defined metric set, well-determined functional implications and system properties can be identified and compared.

For example, Bharatula et al. [14] defined four conflicting metrics and analysed the inherent trade-off on an activity recognition task: Flexibility, which included estimation of memory demand and μC’s operating frequency, electronic packaging, relative recognition performance, and energy consumption measures. The conflicting nature of the four metrics was highlighted as orthogonal, meaning that optimising all the four metrics at the same time is not feasible. Similar to our work, the authors embedded recognition algorithm characteristics in the trade-off analysis, namely classification accuracy. However, we included execution time ET to highlight dynamic design aspects that appear during runtime. The ET metric linked the algorithmic computational complexity with the hardware μC characteristics in time. Understanding of the system temporal constraints enables DynDSE to leverage dynamic system behaviour for context-adaptivity. Azariardi’s [38] DSE included ET and classification accuracy in the metric set but omitted energy consumption. The authors were able to estimate temporal constraints for SVM processing and investigated how the DSE solution matches with application requirements and free-living user. However, assumptions were needed to compensate for the missing energy consumption (EC) metric. The application of Beretta et al. [17] consisted of a wearable node transmitting sensor data using compressive sensing. Objectives were the node’s energy consumption, the percentage root-mean-square difference (PRD) to approximate the information loss due to compression, the communication delay and the packet error rate (PER) of the radio transmission. The solution space was compared with the one reported by Kumar et al. [39], which optimised only energy consumption and communication delay. Under the same energy consumption and communication delay solution, the PRD and PER were significantly higher. Moreover, Kumar et al. were able to discover only the 2.3% of the solution space with respect to Beretta’s work.

From the above comparison, it appears that the descriptive power of the trade-off analysis in DSE depends on a careful selection of the metrics. Neglecting metrics may result in misleading results. The same conclusion can be drawn with regard to our work. For example, consider Figure 9 and Figure 10: When omitting EC, it may seem that the choice of sampling mode does not affect the system behaviour. As the EC is included in our trade-off analysis, it becomes evident how the uniform sampling mode does not provide any feasible configuration. DSE frameworks that consider hardware-software co-design, in principle, achieve higher system performances as a consequence of the flexibility given by a finer model granularity. For example, Shoaib et al. [40] optimised the individual processing stages of a SVM pipeline by exploring hardware architectures based on custom instructions and coprocessor computations. The authors reported a reduction in energy consumption of almost three orders of magnitude compared to that of a low-power μC, as targeted by our work. The energy consumption metric was computed as the sum of several real measurements related to hardware components involved in the SVM processing stage. The design space solutions included specific hardware to run kernel-based classification in varying contexts. The optimisation potential of hardware-software co-design comes at the cost of an expensive design, which includes custom-made platforms, and design space and metrics definition that rely on hardware-specific knowledge.

Overall, dynamic system configurations have been rarely considered in DSE for wearable systems. The inclusion of data sampling strategies into the design space enabled us to adapt system designs to context. Moreover, memory demand has been infrequently included into the DSE objective set, although memory limitations are common in μCs and represent a bottleneck for embedded recognition algorithm deployment, as evident from Figure 9 and Figure 10. We argue that memory demand should be considered in the design phase.

This work focuses on one typical wearable IoT application in order to derive a detailed analysis of the design space spanned by two retrieval algorithms, three μCs, and two sampling procedures introducing dynamic variations. Nevertheless, we kept the DynDSE design space formalism general, such that a wide variety of other components, system architectures, metrics, and IoT applications could be explored, including other hardware, data, and recognition algorithms. Thus, the DynDSE approach does not depend on the particular application considered nor does the method require modifications for other applications. Rather, we deem it essential to match the DynDSE approach with appropriate sensor data to drive the simulation.

For larger design spaces than the one considered here, DynDSE may require approximate rules. Nevertheless, the exhaustive search deployed here remains a suitable option for coarse design selections before investigating further design variables in subsequent, local explorations.

## 7. Conclusions and Future Work

We introduced a general methodology for multi-objective DynDSE applied to context-adaptive wearable IoT edge devices, which retrieve events from streaming sensor data using pattern recognition algorithms. We provided a formal characterisation of the configuration space given a set of system functionalities, components and their parameters. A constrained optimisation problem was formulated to identify an optimal system configuration according to application-dependent system requirements. The simulation can provide crucial information about the compatibility of system components. The method is particularly suitable to analyse design options at an early stage of the development process, to approximate key system design aspects, e.g., size of wireless battery powered devices, to confirm software and hardware choices under given design constraints, and to review designs under varying data patterns.

Further investigations may consider automated, on-demand resource distribution between functions of an embedded system that incorporates the DynDSE methodology. Dynamic resource management may result in wearable IoT systems that reconfigure themselves at runtime according to dynamic conditions. Furthermore, the increasing ubiquity and interconnection among wearable IoT devices rise concerns about security and privacy, as malicious interactions are more likely to happen. System security objectives could be incorporated into the dynamic optimisation to represent varying privacy concerns. Nevertheless, further research is needed to effectively quantify security and privacy concerns in metrics.

## Figures and Tables

**Table 1:** Our metrics and their relative system requirements. The table also indicates the elements which affect the system requirements. *T* denotes the runtime duration and *m* the data frame length.

							The Requirement Is Imposed by		
	Objective	Abbreviation	Description		Requirement	Value	Task	Battery life	μC
π(X|E,Ω)	$\pi^{1}\left(X|E,\Omega \right)$	P	Precision		$z_{\pi }^{1}$	≥70%,	X		
	$\pi^{2}\left(X|E,\Omega \right)$	R	Recall		$z_{\pi }^{2}$	≥80%	X		
ρ(X|E,Ω)	$\rho^{1}\left(X|E,\Omega \right)$	ET	Ex. time in real-time		$z_{\rho }^{1}$	Real-time: <m	X		X
			Ex. time online			Online: <T	X		X
	$\rho^{2}\left(X|E,\Omega \right)$	EC	Energy Consumption		$z_{\rho }^{2}$	≤52.8mWh	X	X	
	$\rho^{3}\left(X|E,\Omega \right)$	MD	Memory Demand		$z_{\rho }^{3}$	Variable (see Table 5)			X

**Table 2:** Design space for the EMG-monitoring eyeglasses.

Functionality ($\Xi_{\xi }$)	Algorithm ($\Xi_{1}$)	Data Sampling ($\Xi_{2}$)	Microcontroller ($\Xi_{3}$)	Runtime Mode ($\Xi_{4}$)
**Component** ($\epsilon_{\xi ,q}$)	FFT-based ($\epsilon_{1,1}$)	Uniform ($\epsilon_{2,1}$)	PSoC1 M8C ($\epsilon_{3,1}$)	Real time ($\epsilon_{4,1}$)
	WPD-based ($\epsilon_{1,2}$)	Adaptive ($\epsilon_{2,2}$)	TI MSP430F1611 ($\epsilon_{3,2}$)	Online ($\epsilon_{4,2}$)
			ARM CortexM3 ($\epsilon_{3,3}$)	
**Parameter** ($\omega_{\xi ,q,w}$)	Data frame size *m* ($\omega_{1,1,1}$)	
	Data frame size *m* ($\omega_{1,2,1}$)	Context measure’s upper bound $\theta_{h}$ ($\omega_{2,2,1}$)		
	Dim. feature space *d* ($\omega_{1,2,2}$)			

**Table 3:** Analytical breakdown of the two spotting algorithms. Each function *f* belongs to an algorithm stage κ. The variable *m* denotes the length of the window size. For the WPD-based feature extraction, the variable *c* denotes the number of WPD coefficients, *l* denotes the depth level of the WPD decomposition tree, and *d* denotes the dimensionality of the feature space after applying PCA. For spotting, the variable *v* is the number of support vectors. The operations for pre-processing, feature extraction, and spotting are calculated on each sliding window’s instance.

Algorithm	Stage (κ)	Function (f)	N. Arithmetical Operations							Memory Demand	
			**Add**	**Mult**	**Div**	**Root**	**Comp**	**Exp**		**Integers**	**Floats**
Both	Pre-processing	Low-pass filtering	*m*	-	-	-	-	-		-	1
FFT-based	Feature extraction	Standard deviation	3m−1	*m*	2	1	-	-		1	-
		Fast Fourier trasform	$3m\cdot log_{2}m$	$2m\cdot log_{2}m$	-	-	-	-		-	*k*
		Maximum	-	-	-	-	m−1	-		1	-
		L2-norm	d−1	*d*	-	1	-	-		1	-
WPD-based	Feature extraction	WPD	$\sum_{i}^{l}\frac{(m/2^{i})-1}{2+1}\cdot 2^{i}$	$\sum_{i}^{l}\frac{(m/2^{i})+1}{4}\cdot 2^{i}$	-	-	-	-		-	4+d
		PCA	d·(c−1)	d·c	-	-	-	-		-	c·d
		L2-norm	d−1	*d*	-	1	-	-		1	-
Both	Spotting	Kernel SVM	v+1	2v	-	-	1	-		*v*	v·(d+1)
		+ Radial basis kernel	v·(2d−1)	v·(d+1)	-	-	1	*v*		-	1

**Table 4:** Analytical breakdown of the context-adaptive sampling algorithm. The variable *n* denotes the number of samples taken during the *n*-shots measure, and *g* denotes the number of channels. The function is executed at any *n*-shot measure.

Function (f)	N. Arithmetical Operations						Memory Demand	
	**Add**	**Mult**	**Div**	**Root**	**Comp**	**Exp**	**Integers**	**Floats**
Context measure	2n·g	-	2	-	n−1	-	-	1
Response output	4	1	1	-	-	-	-	4
Attention time	-	-	-	-	2	-	-	2

**Table 5:** Number of machine cycles $n_{x}^{cyc}$ for the arithmetical operation *x* and memory specifications for the considered μCs.

		Number of Machine Cycles per Operation							Memory spec. [kB]	
		$n_{\mathrm{Add}}^{cyc}$	$n_{\mathrm{Mult}}^{cyc}$	$n_{\mathrm{Div}}^{cyc}$	$n_{\mathrm{Root}}^{cyc}$	$n_{\mathrm{Comp}}^{cyc}$	$n_{\mathrm{Exp}}^{cyc}$		ROM/ Flash	RAM
μC	PSoC1 M8C	544	560	912	1344	80	2672		32	2
	TI MSP430F1611	177	153	405	668	37	334		48.25	10
	ARM CortexM3	60	50	80	380	12	210		512	96

**Table 6:** Component electrical characteristics to estimate the EC and MD measures via simulation. All values were extracted from datasheets. The following symbology is used. $I_{\mathrm{act}}$: current consumption in active mode; $I_{\mathrm{stb}}$: current consumption in stand-by mode; V: voltage; ν: frequency; Res.: resolution; Cap.: capacity; $M_{b}$: data block’s size; $I_{\mathrm{write}}^{m}$: current consumption for memory writing; $t_{\mathrm{write}}$: time for writing a data block.

Functionality	Component	Electric Characteristics								
								Memory Write Operation		
		$I_{\mathrm{act}}\left[\mathrm{mA}\right]$	$I_{\mathrm{stb}}\left[\mathrm{mA}\right]$	*V*	ν[MHz]	Res.[bit]	Cap.[mWh]	$M_{b}$[Byte]	$I_{\mathrm{write}}^{m}\left[\mu A\right]$	$t_{\mathrm{write}}\left[\mathrm{ms}\right]$
μC ($\Xi^{3}$)	PSoC1 M8C ($\epsilon^{3,1}$)	8.0	0.025	3.3	24	8	-	64	619.5	1.5
	TI MSP430F1611 ($\epsilon^{3,2}$)	0.57	0.05	3	8	16	-	60	2300	23.0
	ARM CortexM3 ($\epsilon^{3,3}$)	7.0	0.55	3.3	48	32	-	256	500	3.28
Data Sampling ($\Xi^{2}$)	EMG sensing	4.0	0.008	3.3	$256\times 10^{-6}$	-	-			
Power supply	Li-Ion polymer battery	-	-	3.7	-	-	925			

## Abbreviations

The following abbreviations are used in this manuscript:

μC	Microcontroller
BLE	Bluetooth Low Energy
CL	Communication latency
DSE	Design space exploration
EC	Energy consumption
ECG	Electrocardiography
EEG	Electroencephalography
EMG	Electromyography
ET	Execution time
FFT	Fast Fourier transform
FPGA	Field Programmable Gate Array
GPU	Graphics Processing Unit
IoT	Internet of things
IP	Intellectual Property
LOPO	Leave-one-participant-out
MPS	Maximum payload size
ocSVM	One-class support vector machines
P	Precision
PCA	Principal component analysis
R	Recall
SVM	Support vector machines
WPD	Wavelet packet decomposition
