# Correction: Schiboni et al. DynDSE: Automated Multi-Objective Design Space Exploration for Context-Adaptive Wearable IoT Edge Devices. *Sensors* 2020, *20*, 6104

**DOI:** 10.3390/s22186808

**Published:** 2022-09-08

**Authors:** Giovanni Schiboni, Juan Carlos Suarez, Rui Zhang, Oliver Amft

**Affiliations:** Digital Health, Friedrich-Alexander-Universität Erlangen-Nürnberg (FAU), 91052 Erlangen, Germany

Due to formal academic regulations, the affiliation of the university has been amended, and an “Acknowledgements” section has been added to the original publication [1].

Instead of “FAU Erlangen-Nürnberg”, it should be written as “Friedrich-Alexander-Universität Erlangen-Nürnberg (FAU)”.

The “Acknowledgements” section has been added at the bottom of the paper, just before the “Conflict of Interest”. The content now reads as follows:

**Acknowledgements:** The present study was performed in (partial) fulfillment of the requirements for obtaining the degree “Dr. rer. biol. Hum”.

This correction was approved by the Academic Editor. The original publication has also been updated.
